# Autophagy-like processes are involved in lipid droplet degradation in *Auxenochlorella protothecoides* during the heterotrophy-autotrophy transition

**DOI:** 10.3389/fpls.2014.00400

**Published:** 2014-08-14

**Authors:** Li Zhao, Junbiao Dai, Qingyu Wu

**Affiliations:** MOE Key Laboratory of Bioinformatics, School of Life Sciences, Tsinghua UniversityBeijing, China

**Keywords:** Atg8, Atg4, autophagic vacuole, lipid droplet, microalgae

## Abstract

Autophagy is a cellular degradation process that recycles cytoplasmic components in eukaryotes. Although intensively studied in yeast, plants, and mammals, autophagy in microalgae is not well understood. *Auxenochlorella protothecoides* is a green microalga that has the ability to grow either autotrophically when under light or heterotrophically when in media containing glucose. The two growth modes are inter-convertible and transition between them is accompanied by drastic changes in morphology and cellular composition; however, the mechanisms underlying these changes are unknown. In this study, we identified autophagy-related genes and characterized their roles in the degradation of lipid droplets during the heterotrophy-to-autotrophy (HA) transition in *A. protothecoides*. Most of the proteins constituting the eukaryotic “core machinery” were conserved in *A. protothecoides*. Two proteins, Atg4 and Atg8, were further investigated. *A. protothecoides ATG4* was cloned from a cDNA library and expressed within yeast, and was able to functionally restore the autophagy pathway in *atg4*Δ yeast during nitrogen starvation. Furthermore, Atg8, which displayed high sequence identity with its yeast homolog, was able to conjugate to phosphatidylethanolamine (PE) *in vitro* and was recruited to the phagophore assembly site in yeast. We also identified a C-terminal glycine residue, G118, that was the cleavage site for Atg4. Finally, we used confocal and transmission electron microscopy to reveal that autophagic-like vacuoles were detectable in algal cells during the HA transition. Our data suggested that the lipid droplets in heterotrophic cells were engulfed directly by the autophagic-like vacuole instead of via autophagosomes.

## Introduction

All organisms need to adjust their growth and metabolic status according to changes in environmental and nutritional cues. Some organisms, named mixotrophs, have the ability to grow either autotrophically via photosynthesis or heterotrophically by utilizing organic carbon sources directly from the environment. Consequently, mixotroph cells display dramatic differences in their morphology and cellular composition under the two growth conditions. *Auxenochlorella protothecoides*, a free-living unicellular alga, grows under autotrophic conditions by forming a large cup-shaped chloroplast within the cell, and the cell largely consists of proteins and carbohydrates (Lu et al., 2013). When switched to heterotrophic conditions (limited nitrogen and abundant glucose), *A. protothecoides* grows rapidly and accumulates large amounts of lipid (>50% of dry cell weight). This lipid has been harnessed for the production of biofuel (Miao and Wu, 2006; Xiong et al., 2010; Lu et al., 2013). The two different growth modes are inter-convertible, and completely depend upon nutrient availability in the growth medium. *A. protothecoides* therefore provides a good model for the study of biogenesis and degradation of chloroplasts and the lipid body.

Autophagy is one of the two major degradative systems that eukaryotes employ for quality control of proteins and organelles (Lilienbaum, 2013). Three types of autophagy have been described so far: macroautophagy, microautophagy, and chaperone-mediated autophagy (CMA) (Mizushima, 2007). Autophagy has been intensively investigated in diverse organisms from the fungal, animal, and plant kingdoms and a large number of AuTophaGy-related (ATG) genes have been discovered (Tsukada and Ohsumi, 1993; Hanaoka et al., 2002; Yang and Klionsky, 2009; Xia et al., 2011). Studies on these ATG genes suggest that the core machinery of autophagy is highly conserved in eukaryotes (Meijer et al., 2007; Xia and Klionsky, 2007; Yang and Klionsky, 2009; Mizushima and Komatsu, 2011; Liu and Bassham, 2012).

Recently, several studies suggested that autophagy participates in the degradation of lipid droplets (LDs) (Singh et al., 2009; Kurusu et al., 2014; van Zutphen et al., 2014). LDs are well-defined organelles, delimited by a single protein-associated membrane, that mainly contain lipid esters, i.e., triacylglycerols (TAGs) and cholesteryl esters (Fujimoto and Parton, 2011). LDs are found in most cell types, including yeast, plant seeds, and adipocytes, but the lipid content is highly variable. In addition to the lipid storage function, LDs are involved in lipid metabolism and homeostasis, and have been implicated in several metabolic diseases. Singh et al. (2009) showed that LDs in mouse hepatocytes were degraded by macrolipophagy (the formation of autolipophagosomes and their delivery to the lysosomes). Similar processes were also reported in plants and fungi. For example, autophagy-deficient mutants in rice exhibited delayed pollen maturation and male sterility as a result of blocked degradation of LDs in tapetum cells (Kurusu et al., 2014). In addition, autophagosome-like double membrane structures and vacuole-enclosed lipid droplets were observed during pollen maturation (Kurusu et al., 2014). In yeast cultured with oleic acid media and in *Magnaporthe grisea* during appressorium development, LDs were taken up by vacuoles in a process resembling microautophagy (Weber et al., 2001; van Zutphen et al., 2014).

Studies on autophagy in microalgae remain limited to date. A few recent studies presented the ultrastructure of autophagic-like vacuoles in some cells. Examined cells include *Dunaliella primolecta* in stationary phase, a photosynthesis-deficient mutant of *Chlamydomonas reinhardtii*, *Micrasterias denticulate* under salt or cadmium sulfate stress, *Dunaliella viridis* during nitrogen starvation, and the diatom *Cyclotella meneghiniana* treated with chlorinated benzenes or chromium (Eyden, 1975; Sicko-Goad et al., 1989; Lazinsky and Sicko-Goad, 1990; Inwood et al., 2008; Affenzeller et al., 2009; Jimenez et al., 2009; Andosch et al., 2012). In addition, *C. reinhardtii* Atg8 (CrAtg8) was identified and used as a specific marker for monitoring autophagy (Perez-Perez et al., 2010, 2012). Furthermore, we previously examined sequenced microalgae genomes and located most of the ATG-related genes described in other organisms. This suggests that the eukaryotic autophagy pathway may be conserved within these ancient unicellular organisms (Jiang et al., 2012).

In this study, we employed genomic analysis to identify ATG genes in *A. protothecoides.* We then conducted genetic and biochemical characterization of two of the autophagy-related genes in the Atg8-conjugating pathway (*ApATG8* and *ApATG4*). In addition, we demonstrated that autophagy was involved in the heterotroph-to-autotroph (HA) transition and provided evidence that lipid droplets in heterotrophic cells were degraded by the central vacuole in autophagy-like processes.

## Materials and methods

### Alga strain and growth conditions

*Auxenochlorella protothecoides sp.0710* was originally obtained from the Culture Collection of Alga at the University of Texas (Austin, USA). The culture media and methods of autotrophic and heterotrophic cells were performed as described by Xiong et al. (2010). For induction of the heterotrophy-autotrophy (HA) transition, cells were cultivated in heterotrophic media containing 30 g/l glucose, 2 g/l yeast extract and 0.1 g/l glycine for 7 days and were then transformed into the autotrophic media supplied with light and aseptic air. The glucose and yeast extract were depleted and the glycine was added at the concentration of 5 g/L in autotrophic media.

### Identification of *ApATG8* and *ApATG4*

The gene sequence annotations of *ApATG8* and *ApATG4* from whole genome data were confirmed using CD-search on NCBI. The multiple sequence alignment and 3D structure prediction were performed using Jalview and Swiss-model, respectively. Alignment of the 3D structure was constructed by PyMOL and phylogenetic analysis was conducted by MEGA4.

### RNA extraction and reverse transcription

Seven-day-old heterotrophic liquid culture (10 ml; ~1.8 × 10^8^ cells/ml) was harvested and frozen in liquid nitrogen for 20 min. Cells were powdered using a mortar and pestle with a liquid nitrogen bath. Total RNA was extracted using Trizol (Invitrogen). For genomic DNA removal, ~1 μg total RNA was treated in a 10 μl reaction containing DNaseI, buffer with MgCl_2_ and nuclease-free water at 37°C for 30 min. The reaction was stopped with the addition of 1 μl of 50 mM EDTA and incubation at 65°C for 10 min. Reverse transcription was performed using a first strand cDNA synthesis kit (Fermentas). Genomic DNA-free template RNA (0.1 μg) was added to a 20 μl reaction containing buffer, oligo(dT)_18_ primers, RNase inhibitor, dNTP mix, and reverse transcriptase. The reaction was incubated at 42°C for 60 min and terminated by incubation at 70°C for 5 min.

### Functional complementation of ApAtg4 in yeast

The ORF of *ApATG4* was inserted into the vector pRS415 under the yeast *ADH1* promoter. The construct was transformed into yeast (*BY4741 MATa his3Δ1 leu2Δ0 met15Δ0 ura3Δ0 atg4::KanMX4 pRS316 [GFP-ATG8 URA3]*) using a standard lithium acetate protocol. Transformants were cultured in SC-uracil-leucine medium to log-phase and stained with FM4-64 for 30 min at 30°C. Cells were chased in the same medium for 1 h at 30°C and then transformed into SD (–N) medium for 4 h. Cells were harvested after nitrogen starvation and observed using a confocal microscope (Leica TCS SP5) with GFP and TRITC filters.

### Cleavage assay of ApAtg8 by Atg4

Three different forms of the ApAtg8 protein [wild type, glycine to alanine mutant (G118A), and glycine deletion mutant (ΔG118)] were fused to the N-terminal of GFP. Coding sequences were linked by overlapping extension PCR and the fusion constructs were inserted into vector pRS415 under the *GAL10* promoter with an N-terminal polyhistidine tag. The construct was transformed into a yeast *atg8* mutant (*BY4741 MATa his3Δ1 leu2Δ0 met15Δ0 ura3Δ0 atg8::KanMX4)* and expression was induced with 2% galactose for 2 h. Yeast cells were subjected to immunoblot with anti-His antibody. For the *in vitro* cleavage assay, the ApAtg8-GFP, ApAtg4, and ScAtg4 sequences were cloned into vector pET21b and expressed in *E. coli*. Cells were homogenized by sonication. The reaction was conducted as previously described (Kirisako et al., 2000). The processing of ApAtg8 was detected by western blot using anti-GFP antibody.

### *In vitro* lipidation of Atg8

Phospholipids for liposome preparation were purchased from Avanti Polar Lipids. Lipid mixtures consisting of 1-palmitoyl-2-oleoylphoshatidylcholine (POPC; 80%) and dioleoylphosphatidylethanolamine (DOPE; 20%) were prepared from stocks dissolved in chloroform. After transfer to a glass tube, the mixture was dried to a thin film under a stream of nitrogen. Samples were dried further in a desiccator under vacuum for more than 6 h to remove the traces of organic solvent. The resulting lipid film was hydrated in a buffer consisting of 20 mM Tris-HCl (pH 7.5) and 150 mM NaCl at room temperature for 1 h to produce large multilamellar vesicles (MLVs). MLV suspensions were then disrupted by several freeze-thaw cycles (A single freeze-thaw cycle consisted of freezing for 3 min at liquid nitrogen temperature (−196°C) and thawing for 3 min in a water bath at 42°C). The lipid suspension was then extruded through two polycarbonate membranes (Whatman) of 400 nm pore size using the Avanti Mini-Extruder at room temperature. Liposomes were stored at 4°C.

*In vitro* lipidation of Atg8 was conducted using purified Atg3, Atg5, Atg7, Atg8, Atg12, and liposomes. The ApAtg8 protein was purified using HIS-Select Nickel Affinity Gel (Sigma). Reactions were performed as previously described (Ichimura et al., 2004). To distinguish Atg8 and Atg8-PE, the reaction products were subjected to 13.5% urea-SDS-PAGE and stained with Coomassie blue.

### Co-localization of Atg8 with Ape1

*GFP-ApATG8* and *Ape1-mCherry* were inserted into pRS416 and pRS415, respectively, under the control of the *ADH1* promoter. Yeast *atg8*Δ cells were co-transformed with these two plasmids. Transformants were cultured to log-phase in SC-uracil-leucine medium at 30°C, washed three times, and resuspended in SC-uracil-leucine medium containing 0.2 μg/ml rapamycin for 1 h at 30°C. Cells were harvested and observed using a Zeiss710META microscope using eGFP and mCherry filters.

### Monodansylcadaverine and nile red staining

For monodansylcadaverine and Nile red staining, algal cells were harvested and washed with 100 mM PBS (pH6.8) three times. Cells were stained for 10 min with 0.5 mM monodansylcadaverine dissolved in water (Sigma-Aldrich) at room temperature or with 1 μg/ml Nile red dissolved in DMSO (Genmed Scientifics) at 37°C. Cells were then washed with PBS three times to remove excess fluorescent dyes. Cells were observed using a Zeiss710META microscope with dansylcadaverine, Nile red, and chlorophyll A filters. The MDC-incorporated structures were excited at 405 nm and detected at 451–539 nm. Fluorescence intensity was measured using ImageJ. The original images of HA cells stained with MDC were transformed into 8-bit images and cellular regions were outlined using the threshold tools of ImageJ. Integrated density (IntDen) and area were measured. The final average fluorescent intensity per cell was calculated as IntDen/Area. Approximately 100 cells were measured.

### Determination of total lipid and chlorophyll content in algal cells

Algal cells were harvested, washed twice with distilled water, and lyophilized. Lipid in algal powder was transesterified into biodiesel with sulfuric acid and then measured using gas chromatography as previously described (Xiong et al., 2010). For chlorophyll analysis, algal powder was boiled in DMF (N, N-dimethylformamide) (Amresco) for 5 min for chlorophyll extraction, followed by measurement of OD_663_ and OD_645_ using UV/visible spectrophotometry (Pharmacia Biotech Ultrospec 2000, Sweden) as described previously (Xiong et al., 2010).

### Transmission electron microscopy

*A. protothecoides* cells were fixed in 2.5% glutaraldehyde (pH6.8) for 12 h at 4°C. Samples were prepared as described by Jiang et al. (2012). Cells were imaged using a transmission electron microscope (Hitachi, Japan).

## Results

### Most of the core protein machinery for autophagy is conserved in *A. protothecoides*

**Figure 1:**
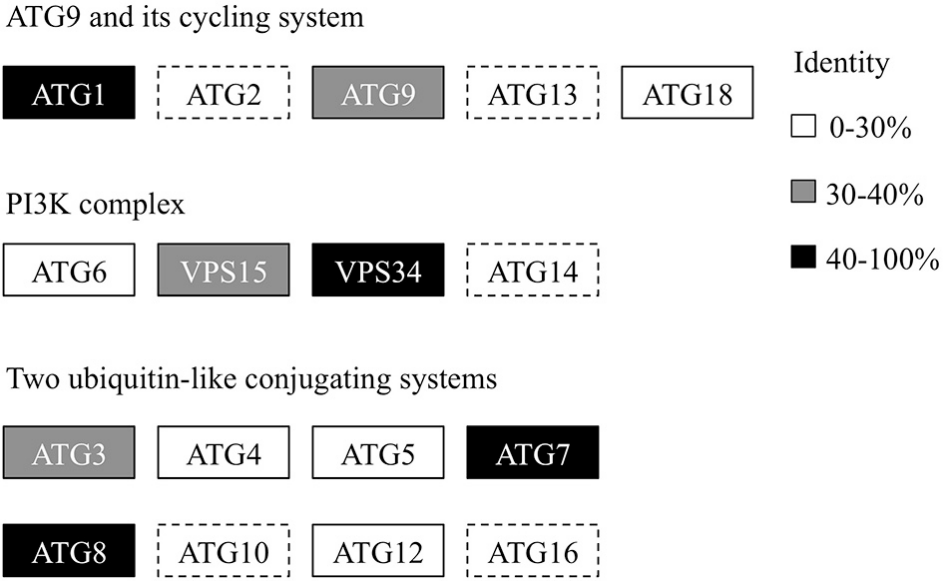
**Genome-wide identification of the autophagy core machinery genes in *A. protothecoides***. Dotted lines indicate missing genes. Boxes show the yeast ATG homologs detected in *A. protothecoides*. The identity level of each gene is indicated by shading.

Recently, we generated the complete genome sequence of *A. protothecoides* (Gao et al., 2014) and identified several hypothetical genes that were annotated as homologs of ATG genes. Next, we conducted a genome-wide protein BLAST analysis using yeast ATG proteins as query sequences. Each identified candidate was subjected to the CD-search at the National Center for Biotechnology Information (NCBI) data bank to confirm the presence of conserved autophagy protein domains. Eventually, we found that most of the identified autophagy-related genes (12 of 17) could be assigned to one of the core machineries that participated in different stages of autophagy. The assigned core machineries included membrane initiation, elongation, closure, and fusion with vacuoles, suggesting that *A. protothecoides* possessed the molecular components needed for autophagy induction. Unlike plants, only a single copy of each ATG gene was identified in the *A. protothecoides* genome. As shown in Figure 1, most of the putative genes had moderate but substantial identities (20–80%) with the yeast homologs. All the identified proteins contained conserved ATG domains (data not shown). Notably, all of the components of the Atg8-conjugating pathway (Atg8, Atg4, Atg5, Atg7, Atg3, and Atg12) were identified in the *A. protothecoides* genome, suggesting that the pathway was likely to be active in this alga.

### Identification of ApAtg8 and ApAtg4

**Figure 2:**
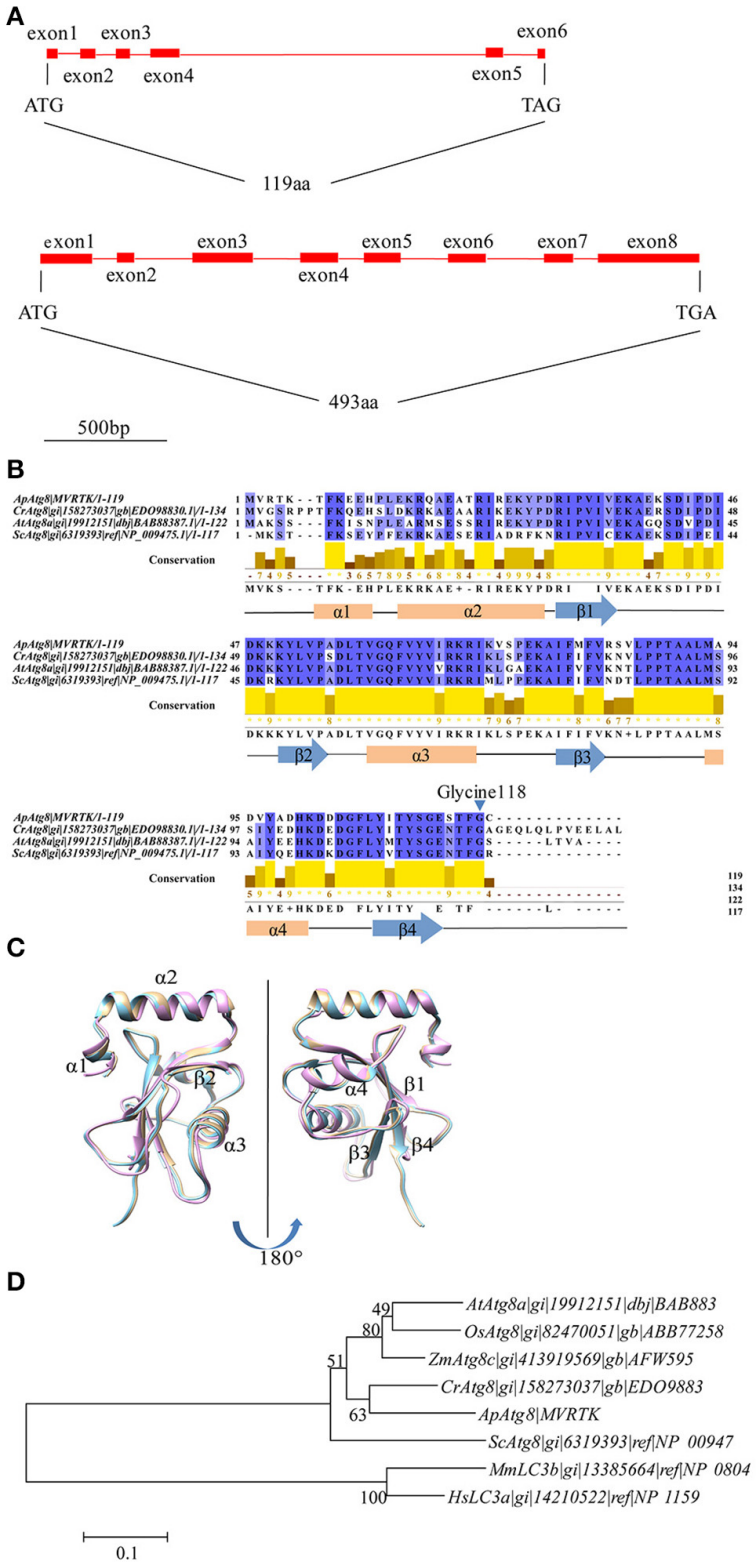
**Identification of *ApATG8* and *ApATG4*. (A)** Genomic structure of *ApATG8* (top panel) and *ApATG4* (bottom panel). Filled boxes indicate exons and straight lines represent introns. **(B)** Multiple sequence alignment of Atg8 proteins. Conservation indexes are shown as yellow numbers. A conserved C-terminal glycine residue at position 118 is indicated with an arrowhead. *Cr*, *Chlamydomonas reinhardtii*; *Ap*, *Auxenochlorella protothecoides*; *At*, *Arabidopsis thaliana*; *Sc*, *Saccharomyces cerevisiae*. **(C)** Overlay of the tertiary structures of ApAtg8 (wheat), yeast Atg8 (cyan), and human LC3 (pink). **(D)** Unrooted phylogenic tree of Atg8 proteins constructed using the Neighbor-Joining method. The branch numbers on the nodes indicate the bootstrapping values. *At*, *Arabidopsis thaliana*; *Os*, *Oryza sativa*; *Zm*, *Zea mays*; *Cr*, *Chlamydomonas reinhardtii*; *Ap*, *Auxenochlorella protothecoides*; *Sc, Saccharomyces cerevisiae*; *Mm*, *Musmusculus*; *Hs*, *Homo sapiens*.

To investigate whether an active autophagy pathway was present in *A. protothecoides*, we focused on characterization of two key components: *ApATG4* and *ApATG8.* The putative *ApATG8* and *ApATG4* genes were amplified from a cDNA library using gene-specific primers (Supplementary Table 1) and were cloned and sequenced. DNA sequence alignments between the cloned cDNA and the genomic DNA (gDNA) sequence revealed the gene structures of *ApATG8* and *ApATG4* (Figure 2A). The *ApATG8* gene spanned approximately 2 kb of the genome and contained 6 exons and 5 introns. The *ApATG4* gene was about 2.6 kb in length and had 8 exons and 7 introns. These gene structures closely resembled the structures of the plant homologs. The ApAtg8 protein also had a considerable degree of identity (~73%) with the yeast homolog. Several C-terminal Atg8 protein sequences from *A. protothecoides* and model organisms were aligned (Figure 2B). This analysis indicated that the C-terminal glycine residue of Atg8 at position 118, which is modified by Atg4 during autophagy induction, was conserved. The predicted ApAtg8 protein structure contained four α-helices and four β-sheets and was consistent with the reported secondary structures of the yeast and human homologs. The predicted tertiary structure of ApAtg8, which consisted of an N-terminal α-helix domain and a C-terminal ubiquitin-like core (Figure 2C), was similar to yeast Atg8 (resolved by NMR) and human LC3 (resolved by crystallization). Phylogenetic analysis of Atg8 proteins from *A. protothecoides* and model organisms indicated that ApAtg8 had the closest relationship to Atg8 from *C. reinhardtii*, which corresponded to the 16s rRNA phylogeny (Figure 2D). Taken together, these data indicate that Atg8 is conserved between eukaryotes.

### ApAtg4 can functionally replace Atg4 in yeast

**Figure 3:**
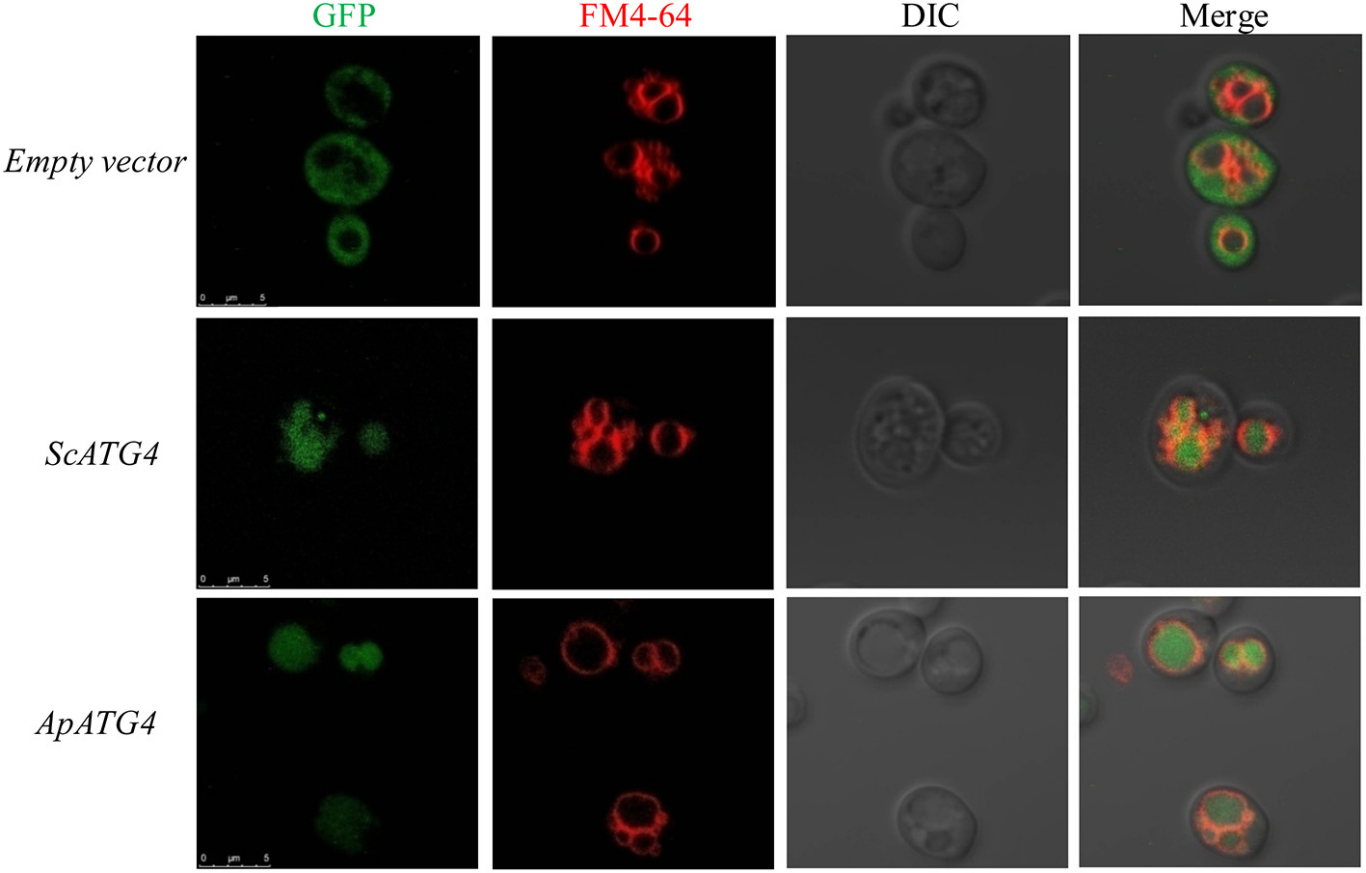
**Functional complementation of ApAtg4 in *atg4* mutant yeast**. *ApATG4* cDNA was cloned and expressed in a yeast *atg4* mutant in a GFP-Atg8 background. The yeast *ATG4* (*ScATG4*) and the empty vector were used as positive and negative controls, respectively. Log-phase cells were transformed into nitrogen starvation SD (–N) medium for 4 h. The yeast vacuolar membrane was stained by FM4-64. DIC, Differential interference contrast microscopy images. Scale bar, 5 μm.

To confirm the function of the ApAtg4 protein, a complementation experiment was conducted in yeast. GFP-Atg8 was used as a marker of autophagy. The ApAtg4 protein was expressed in a yeast strain containing GFP-tagged Atg8 and a complete deletion of *ATG4* (*atg4*Δ). As shown in the top panel of Figure 3, Atg8 was not processed or translocated to the vacuole upon nitrogen starvation when an empty vector was supplied (i.e., no ApAtg4). Localization of GFP-tagged Atg8 was restored upon expression of yeast Atg4 from a plasmid containing *ScATG4* (middle panel). Localization of GFP-tagged Atg8 was similarly restored when *ApATG4* was expressed (bottom panel). This indicated that ApAtg4 could functionally replace yeast Atg4 and restore the autophagy pathway under conditions of nitrogen starvation. ApAtg4 is therefore the homolog of yeast Atg4 and the two proteins probably perform similar functions during autophagy in their respective organisms.

### ApAtg8 is cleaved by Atg4 protease *in vitro* and *in vivo*

**Figure 4:**
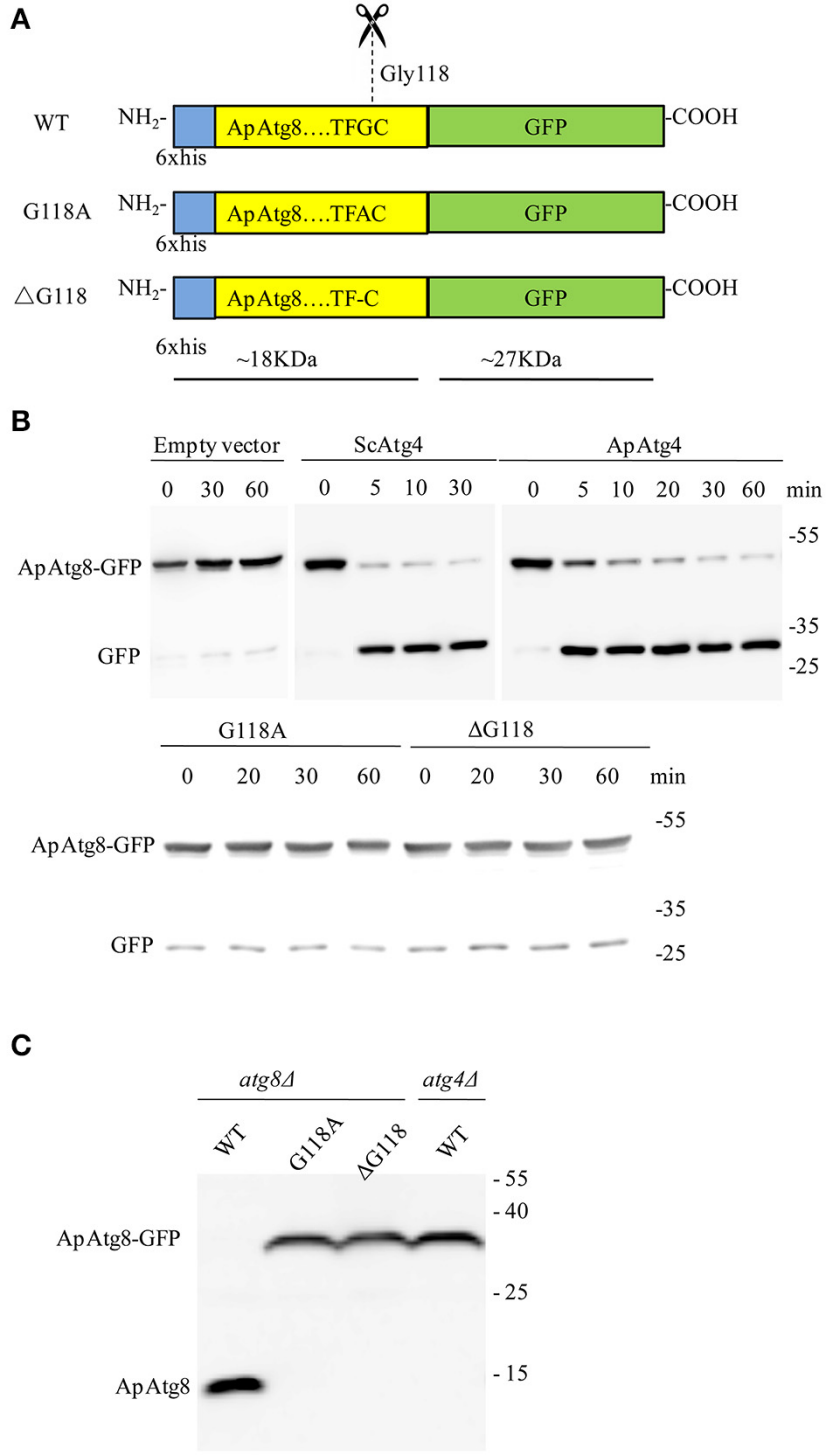
**ApAtg8 is processed by Atg4 at the C-terminal Gly-118 residue. (A)** Diagrams of His-tagged ApAtg8-GFP (WT), ApAtg8^G118A^-GFP (G118A) and ApAtg8^ΔG118^-GFP (ΔG118). **(B)** Assay for cleavage of ApAtg8 by ApAtg4 *in vitro*. *E. coli* cells expressing WT (top panel), G118A and ΔG118 (bottom panel) ApAtg8-GFP were homogenized and the total cell lysates were mixed with ApAtg4 lysate. Reactions were incubated at 30°C for the indicated times. Yeast Atg4 and empty vector were used as the positive and negative controls, respectively. The mixture was subjected to western blot using anti-GFP antibody. **(C)** Cleavage assay in yeast. WT, G118A, and ΔG118 Atg8 were expressed in a yeast *atg8* mutant (lanes 1, 2, and 3) or an *atg4* mutant (lane4). Cells were harvested after nitrogen starvation for 4 h. Total lysates were subjected to western blot using anti-His antibody.

ApAtg4 functioned as a protease and was able to functionally replace yeast Atg4. We therefore hypothesized that ApAtg8 would be processed by cleavage at the C-terminal glycine residue as in yeast Atg4. As mentioned above, a highly conserved glycine residue was located at amino acid position 118 (G118) of ApAtg8, and this was the likely target site for Atg4. To test this possibility, we constructed a reporter protein by fusing ApAtg8 with an N-terminal polyhistidine tag and a C-terminal GFP tag. In addition, plasmids were generated containing one of two mutant *ApATG8* genes, one of which encoded a glycine to alanine substitution (G118A) and one of which encoded a glycine deletion (ΔG118) (Figure 4A). Fusion proteins and Atg4 from yeast (ScAtg4) and *A. protothecoides* (ApAtg4) were expressed in *E. coli*. An *in vitro* Atg8 cleavage assay was performed by mixing the whole-cell extracts from Atg8-expressing bacteria with whole-cell extracts from bacteria expressing ScAtg4, ApAtg4, or empty vector. Western blots were performed using anti-GFP antibody to detect free GFP (Figure 4B). Minimal free GFP was observed in the absence of Atg4. Free GFP was observed in increasing amounts with time when ApAtg8-GFP was incubated with Atg4 from yeast or *A. protothecoides*. The amount of ApAtg8-GFP fusion protein decreased concurrently. Liberation of free GFP did not occur when mutated ApAtg8-GFP (G118A or ΔG118) was incubated with ApAtg4, indicating that cleavage of ApAtg8-GFP by Atg4 was dependent on ApAtg8 G118.

Next, we assessed whether Atg4 could cleave ApAtg8 *in vivo*. Fusion constructs were transformed and expressed in a yeast *atg8*Δ strain and western analysis with anti-HIS antibody was used to identify free ApAtg8 protein. As shown in Figure 4C, ApAtg8-GFP was cleaved upon activation of autophagy by nitrogen starvation and a fragment of ~18 kDa was generated. This fragment size corresponded with the size of ApAtg8 protein. Mutant proteins containing either G118A or ΔG118 could not be processed. This indicated that, as with the *in vitro* analysis, G118 was essential for cleavage of ApAtg8. Cleavage was also completely abrogated in *atg4*Δ yeast, which indicated that Atg4 was also essential for processing of ApAtg8. Taken together, our data indicate that ApAtg8 can be cleaved by Atg4 *in vitro* and *in vivo*, and that Atg4 and ApAtg8 G118 are both necessary for processing.

### ApAtg8 can be conjugated to phosphatidylethanolamine *in vitro*

**Figure 5:**
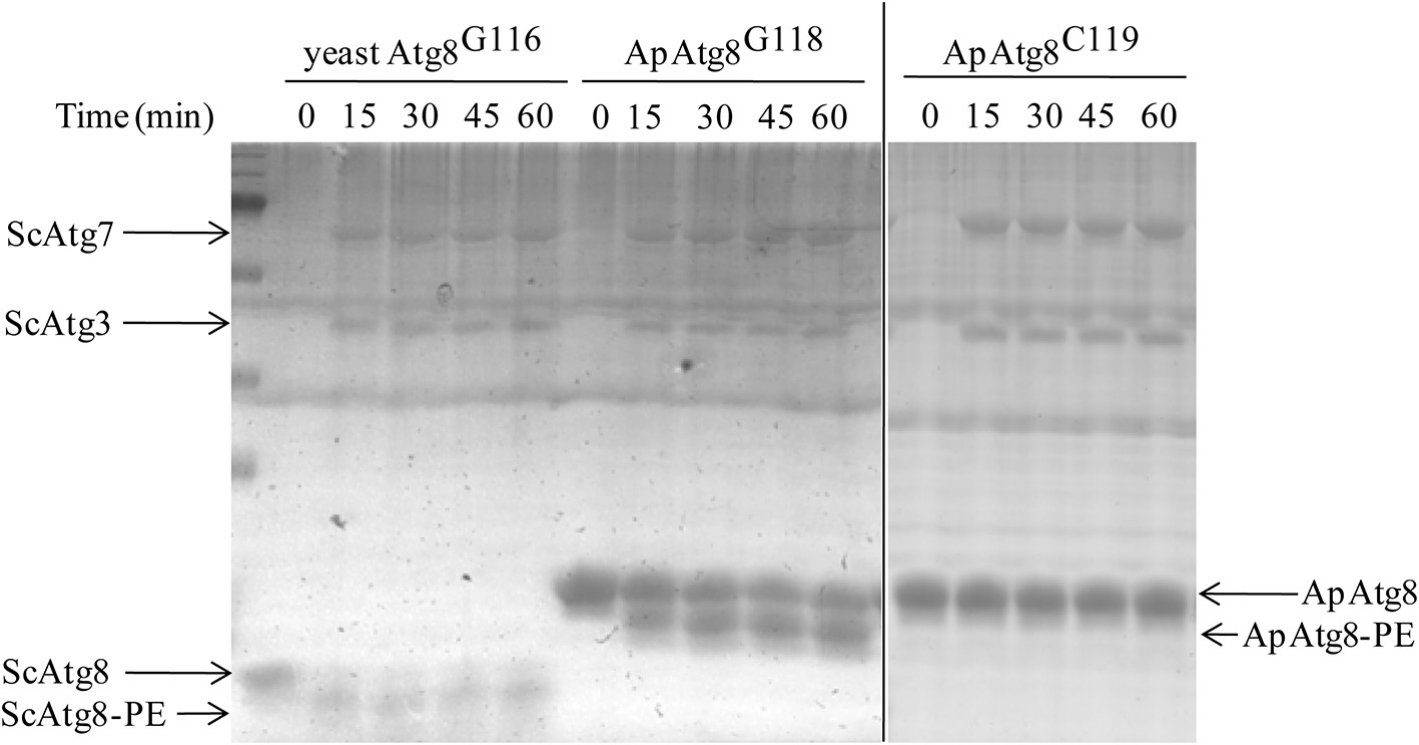
***In vitro* lipidation of ApAtg8**. *In vitro* conjugation reactions were performed at 30°C in the presence of purified yeast Atg3 (ScAtg3), Atg5, Atg7 (ScAtg7), ApAtg8^G118^ (the processed form of ApAtg8 whose C-terminal glycine residue was exposed), and Atg12. Yeast Atg8^G116^ and the nascent from of ApAtg8 (ApAtg8^C119^) were used as positive and negative controls, respectively.

After processing, Atg8 is conjugated to phosphatidyl-ethanolamine (PE) in a process crucial for autophagosome formation known as Atg8 lipidation. The Atg8 conjugation system was reconstituted *in vitro* using purified ATG proteins and liposomes to provide an experimental environment to assess ApAtg8 lipidation. Purified ApAtg8^G118^, with C-terminal glycine exposed, was added to a reaction system containing purified yeast Atg7, Atg3, Atg5-Atg12, and 20% PE liposomes. Yeast Atg8^G116^ was used as a positive control and ApAtg8^C119^ (no exposed C-terminal glycine) was used as a negative control. Urea-SDS-PAGE showed that the nascent form of ApAtg8 was detected as a single ~16 kDa fragment, as expected (Figure 5). A faster-migrating fragment, representing lipidated Atg8, was observed after incubation with ApAtg8^G118^ or yeast Atg8^G116^ for 15 min. No lipidation was detected when the terminal glycine was masked (ApAtg8^C119^). We therefore concluded that ApAtg8 could be conjugated to PE and that the conserved glycine at position mediated conjugation.

### ApAtg8 can be recruited to the phagophore assembly site in yeast

**Figure 6:**
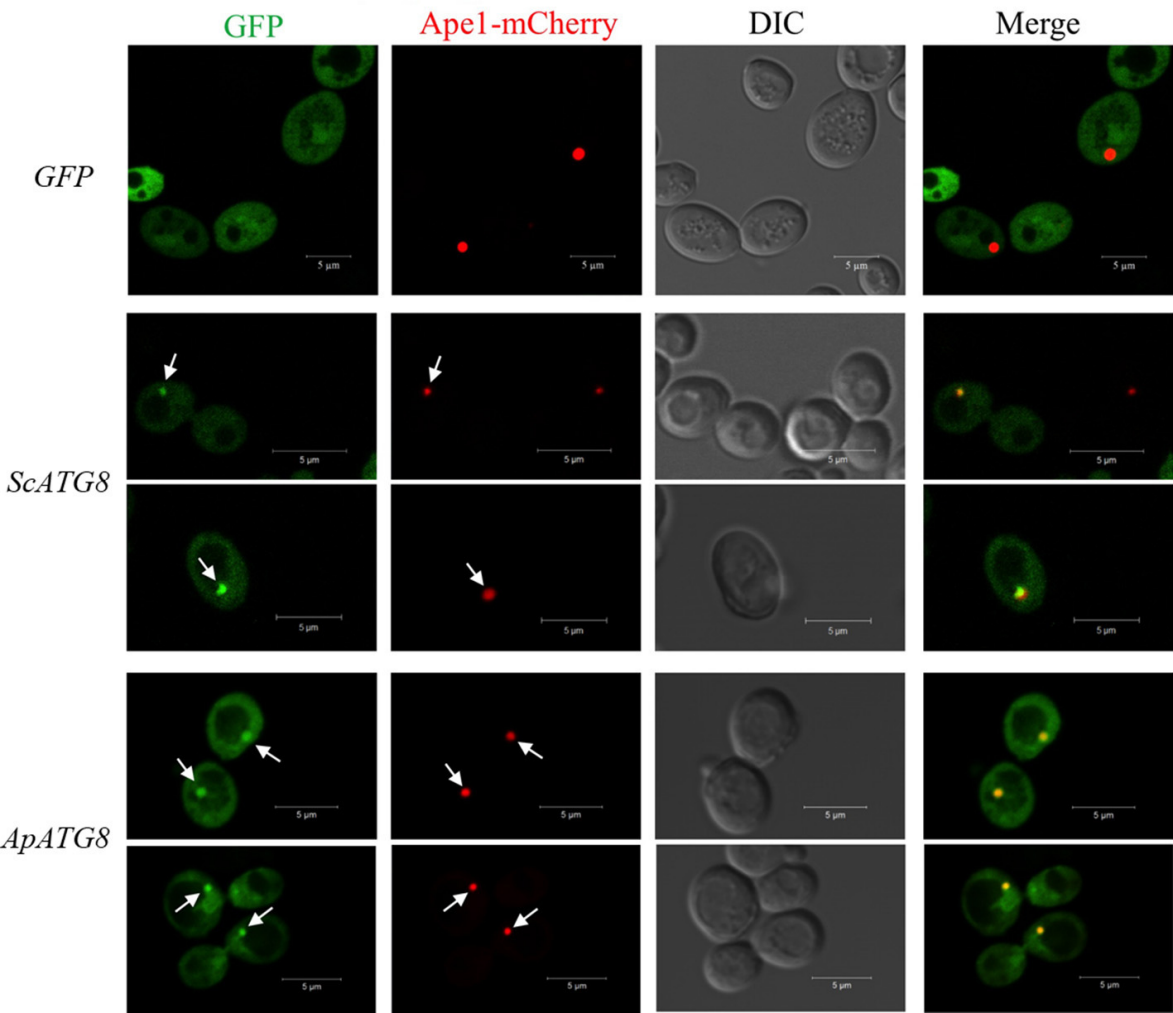
**ApAtg8 co-localizes with Ape1 at the PAS**. *ApATG8* cDNA was expressed in *atg8*Δ yeast. Yeast *ATG8* (*ScATG8*) and the empty vector were used as positive and negative controls, respectively. Cells in log-phase were shifted to SC-uracil-leucine medium containing 0.2 μg/ml rapamycin for 1 h. Arrows indicate the punctate structures where GFP-Atg8 and Ape1-mCherry co-localize. DIC, Differential interference contrast microscopy images. Scale bar, 5 μm.

After lipidation, Atg8 is recruited to the phagophore assembly site (PAS), where the core autophagy machinery was assembled. In yeast, Ape1 is recruited to PAS and is then transported to the vacuole for maturation by the cytoplasm to vacuole targeting pathway (CVT). We wished to determine whether ApAtg8 could be recruited to PAS. Ape1 co-localizes with Atg8 in yeast (van der Vaart et al., 2010) and we therefore examined co-localization of Ape1 and ApAtg8. GFP-labeled ApAtg8 and mCherry-labeled Ape1 were expressed in yeast cells and examined by fluorescence microscopy. GFP-alone was observed as diffuse green fluorescence and Ape1-mCherry was seen as spherical dots. As with yeast Atg8-GFP, ApAtg8-GFP formed punctate structures and co-localized with Ape1 after rapamycin treatment (Figure 6), indicating that ApAtg8 localized to the PAS during induction of autophagy in yeast.

### Degradation of lipid bodies and biogenesis of the photosynthetic system during the HA transition

**Figure 7:**
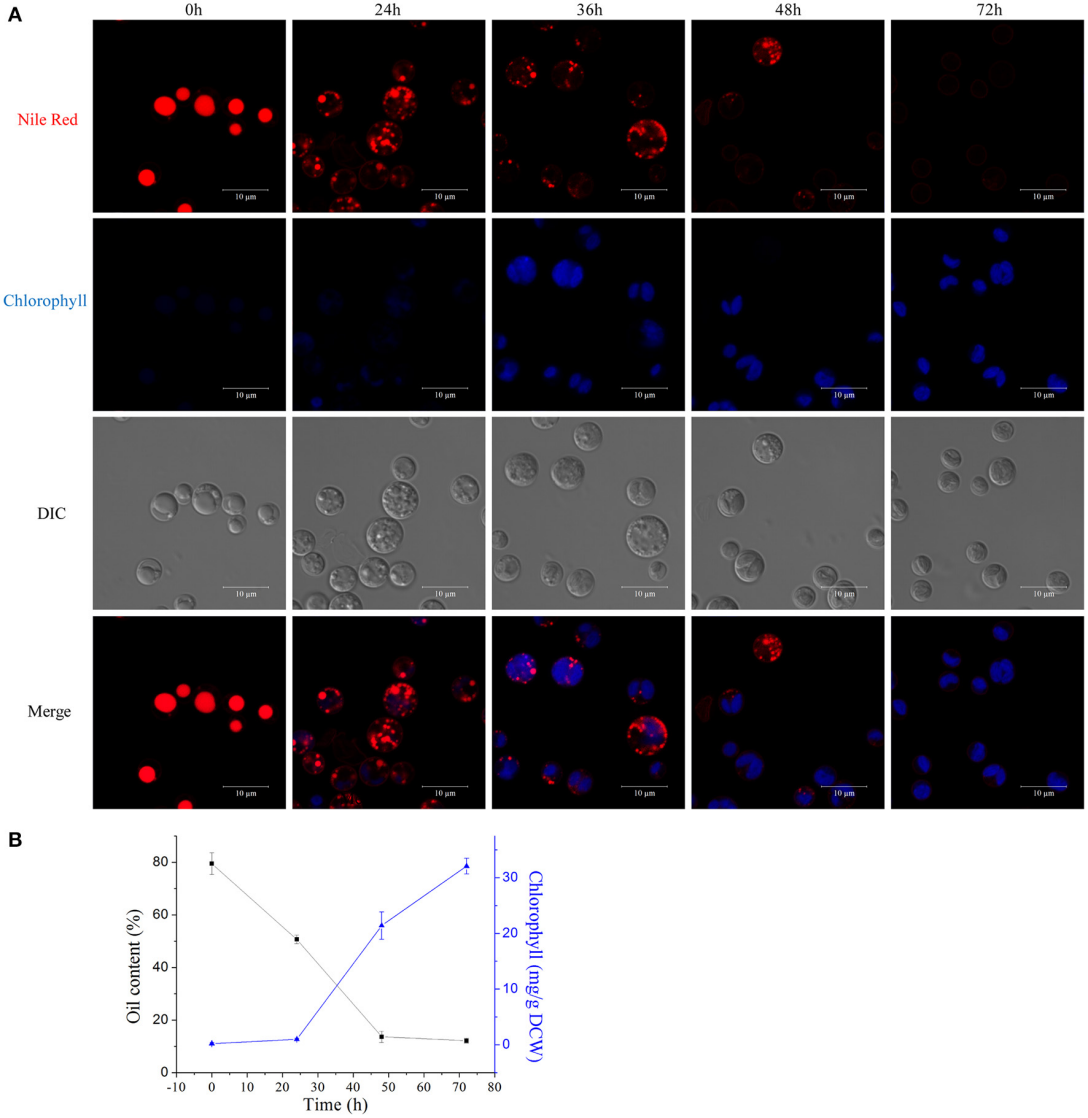
**Degradation of lipids and biogenesis of chloroplasts during the HA transition. (A)** Confocal microscopy images of algal cells during the HA transition. Cells were cultivated in heterotrophic medium for 7 days and then transferred into autotrophic medium for 3 days. LDs were stained using Nile red and chlorophyll auto-fluorescence is shown as a pseudo-color image in blue. DIC, Differential interference contrast microscopy images. Scale bar, 10 μm. **(B)** Determination of total lipid and chlorophyll contents of HA cells. Closed black square, lipid content; closed blue triangles, chlorophyll content.

We examined the morphological and biochemical component changes that occurred during the *A. protothecoides* HA transition. Algal cells were cultivated in heterotrophic media for 7 days, harvested, and used to inoculate autotrophic media (high nitrogen, glucose deprivation). Cells were stained for lipid using Nile red and were examined by confocal microscopy (Figure 7A). Large lipid bodies occupying more than half of the entire cellular space were typically found in heterotrophic cells at the initiation of the HA transition. The lipid bodies subsequently shrank in size and chlorophyll auto-fluorescence emerged during the first 24 h of adaptation to light. Assembly of chloroplastic structures was completed within 72 h. Cup-shaped or spherical chloroplasts were dominant, which suggested that the photosynthetic system was completely reconstructed. Analysis of total cellular lipid and chlorophyll contents confirmed the degradation of lipid and biosynthesis of chlorophyll. The lipid content gradually decreased from ~80% (w/w) at the initiation of the HA transition to 10% (w/w) after 72 h cultivation in autotrophic media. During the same time period, chlorophyll content increased to greater than 30 mg/g dry cell weight (Figure 7B).

### Autophagy induction during the HA transition in *A. protothecoides*

**Figure 8:**
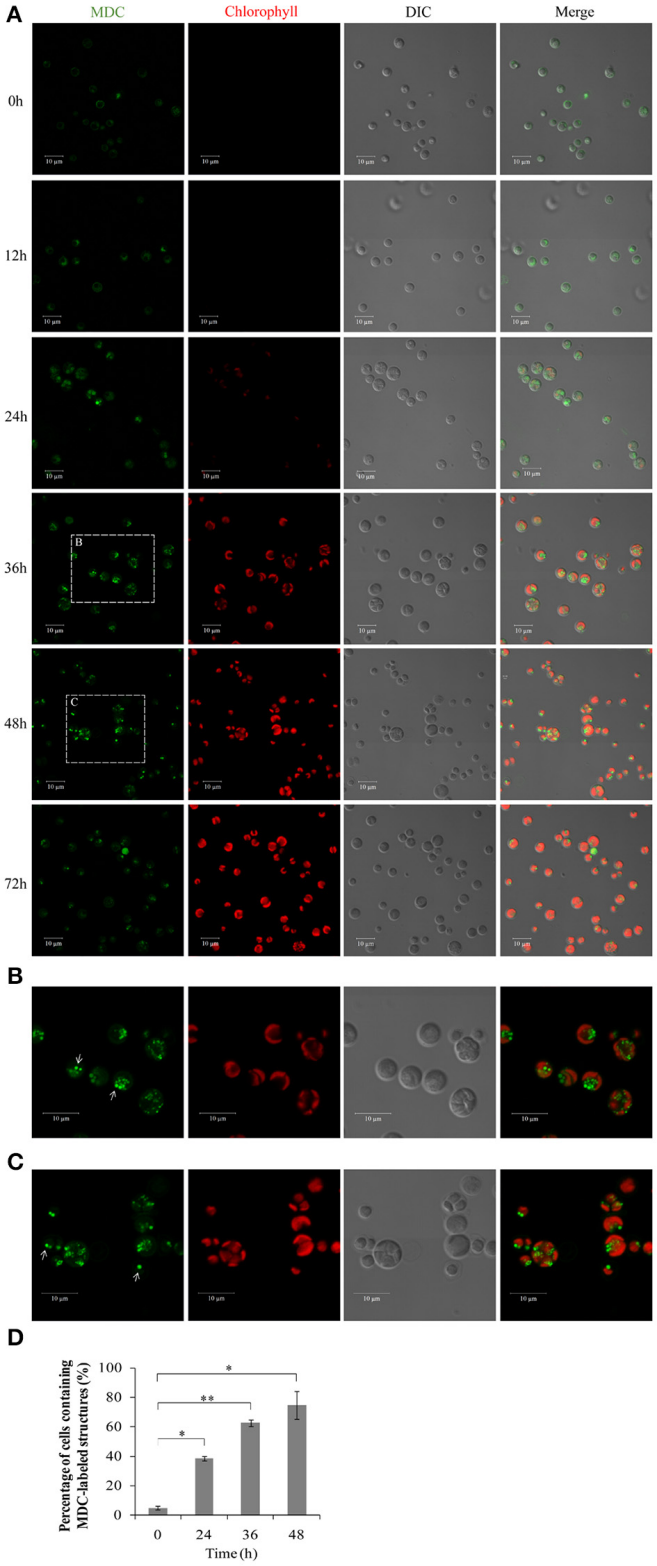
**Confocal microscope images of *A. protothecoides* during the HA transition. (A)** MDC staining of *A. protothecoides*. Seven-day heterotrophic cells were inoculated into autotrophic medium and exposed to light at 28°C. Filtered air was supplied. Cells were harvested at the indicated times, stained with MDC, and observed using a confocal microscope. The MDC structures are shown in green and chlorophyll A is shown in red. Scale bar, 10 μm. **(B,C)** Magnified images of *A. protothecoides* cells from **(A)**. Scale bar, 10 μm. White arrows indicate MDC-stained autophagic bodies. **(D)** Quantification of MDC-stained structures. More than 100 cells were counted each time and the percentage of cells containing MDC-labeled structures was calculated. Error bars represent standard error from two independent experiments. Student's *t*-test was used to determine significant differences (^*^P < 0.03, ^**^P < 0.01).

To investigate whether autophagy is involved in the HA transition, cells at different time points were monitored using monodansylcadaverine (MDC), a fluorescent dye that stains the autophagic structures *in vivo* (Biederbick et al., 1995; Munafo and Colombo, 2001; Contento et al., 2005). Single-dye labeling samples were tested to ensure that bleed-through signal between filters did not occur (data not shown). The intensity of the MDC fluorescent signal increased >5–fold between 0 and 36/48 h (Figure 8A). Several MDC-labeled spherical structures were found in close proximity to the chloroplast envelope (Figures 8B,C). These mobile structures were probably autophagic bodies located randomly in the *A. protothecoides* vacuole. Cells containing MDC-labeled structures were quantitated, and numbers of MDC-positive cells rose during the 48 h after the shift to autotrophic growth. Approximately 15–fold more MDC-positive cells were found at 48 h than at 0 h (Figure 8D; ^*^p < 0.03, ^**^p < 0.01).

**Figure 9:**
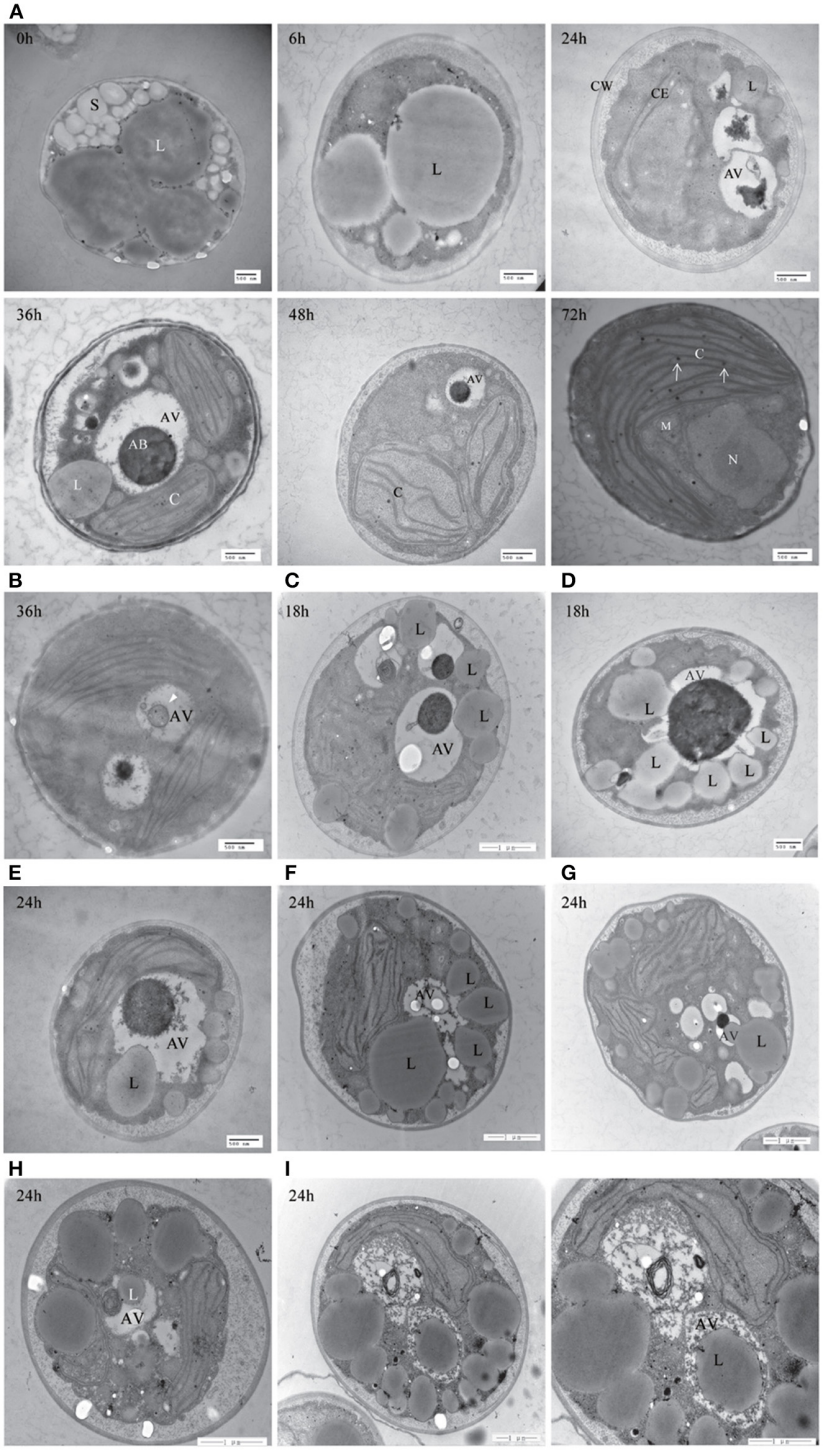
**Transmission electron microscope (TEM) images of *A. protothecoides* during the HA transition. (A)** Heterotrophic cells were resuspended in autotrophic medium for the indicated time and then processed for TEM. C, chloroplast; CE, chloroplast envelope; CW, cell wall; S, starch granules; L, lipid bodies; AV, autophagic vacuoles; AB, autophagic bodies; M, mitochondria; N, nuclear. White arrows indicate plastoglobuli in chloroplasts. **(B)** Representative ultrastructure of autophagic vacuoles in HA cells after 36 h cultivation in autotrophic media. Single-membrane-bound vesicles (white arrowhead) were detected within the vacuoles of HA cells. **(C–I)** Different stages of internalization of the small scattered LDs by autophagic vacuoles in *A. protothecoides* exposed to light for 18 or 24 h during the HA transition. L, lipid bodies; AV, autophagic vacuoles.

To further confirm the presence of autophagic-like vacuoles in *A. protothecoides* during the HA transition, transmission electron microscopy was used to investigate ultrastructural changes within algal cells during transition. As shown in Figure 9A, the lipid bodies and starch granules that had accumulated in heterotrophic cells were degraded and the cup-shaped chloroplast was regenerated during the HA transition. This was consistent with the confocal microscopy observations. Autotrophic cells utilized plastoglobuli for lipid storage in chloroplasts (Figure 9A, white arrows). Electron-dense spherical structures and single-membrane-bound vesicles (~0.3–1 μm in diameter) that resembled autophagic bodies were typically detected in the vacuoles of HA cells subjected to autotrophic growth for 36 and 48 h but were absent within cells exposed to 0 h of light (Figures 9A,B). Smaller autophagic vacuoles generated within one single cell early in the HA transition subsequently fused into one large autophagic vacuole (Figure 9A). These data suggest that autophagy was induced during the HA transition. Instead of the large lipid bodies observed in the heterotrophic cells at 0 h, a number of small lipid droplets (LDs) were seen in the proximity of the central vacuole during the HA transition. These LDs, which were delimited by a single membrane, tended to be internalized by the larger (1.5 μm diameter) autophagic vacuole (Figures 9C–E). The different stages of LD internalization by autophagic vacuoles are shown in Figures 9F–I. A minority of LDs that were relatively large partially extruded into the autophagic vacuole (Figure 9G). Figures 9H,I show LDs completely engulfed by autophagic vacuoles. To further confirm the autophagic degradation of lipid bodies in *A. protothecoides* during HA transition, cells were double-labeled with Nile red and MDC and examined with confocal microscopy. Lipid bodies in a number of cells exposed to light for 24–36 h co-localized with MDC-labeled structures, demonstrating that the dense bodies within vacuoles were likely to be LDs (Supplementary Figure 1). No double membrane structures consistent with lipophagy were detected, indicating that the degradation of LDs during HA transition in *A. protothecoides* was probably a microautophagy-like process.

## Discussion

Autotrophic and heterotrophic *A. protothecoides* undergo different growth patterns that result in diverse subcellular structures and chemical composition (Miao and Wu, 2006; Xiong et al., 2010; Lu et al., 2013). The majority of the cellular space in heterotrophic cells comprises large lipid droplets (~1–2 μm) and starch granules and photosynthetic chloroplasts are completely absent. The neutral lipid content varies greatly between heterotrophic (above 50%) and autotrophic (~10%) cells. Algal cells have the ability to undergo HA transition, in which lipid droplets and starch granules diminish and the large cup-shaped chloroplast is regenerated. However, the mechanisms underlying the HA transition remain obscure. In this study, we demonstrated that autophagy-related genes in the ATG8-conjugating pathway were conserved in *A. protothecoides.* We also showed that an autophagy-like process was involved in the degradation of LDs during the HA transition. *A. protothecoides* may therefore serve as a useful study model for the metabolism and dynamics of lipid droplets and chloroplasts.

### Conservation of autophagy-related genes in *A. protothecoides*

Previous studies revealed that autophagy-related pathways evolved in eukaryotes for the chambered-protease degradation of proteins and organelles (Hughes and Rusten, 2007). *A. protothecoides* is a eukaryotic green alga derived from primary endosymbiosis (Keeling et al., 2005). In this study, we performed a genome-wide *in silico* study of the autophagy machinery in *A. protothecoides* and provided evidence for the existence of most autophagy-related genes. This is consistent with our previous survey of autophagy-related genes in seven different microalgae (Jiang et al., 2012). These data suggest that autophagy is an evolutionarily ancient process.

We failed to identify several core autophagy components (Figure 1). As canonical autophagy genes were mainly identified in model organisms, such as yeast, one possible explanation is that the related autophagy pathways might not exist in *A. protothecoides*. Alternatively, these components may lack sufficient sequence conservation with their yeast counterparts to allow identification. Finally, some species-specific autophagy factors or pathways may exist that would compensate for the missing canonical components. In support of the last hypothesis, recent genetic screens of autophagy-deficient mutants in *Caenorhabditis elegans* reveal several uncharacterized metazoan-specific genes required for macroautophagy, including *epg-2*,*-3*,*-4*, and -*5* (Tian et al., 2010). Further investigation will be required to test this possibility.

### How do lipid bodies in heterotrophic algal cells degrade?

Previous studies reveal that LDs perform functions other than lipid storage. LDs are dynamic multifunctional organelles that are involved in various physiological pathways, including membrane synthesis, viral replication, protein degradation, and energy production (Walther and Farese, 2012). Disorders in the metabolism of LDs, particularly those involving excessive LD storage in mammalian tissues, are related to several diseases such as diabetes and atherosclerosis. LDs are hydrolyzed in a process called lipolysis by a series of cytosolic lipases such as adipose triglyceride lipase (ATGL), hormone sensitive lipase (HSL) in mammals, and Tg13& Tg14 in yeast (Fujimoto and Parton, 2011). More recently, autophagy is shown to contribute to the breakdown of LDs for the maintenance of lipid homeostasis in mouse liver, plant seeds, and fungi (Singh et al., 2009; Kurusu et al., 2014; van Zutphen et al., 2014).

In this study, we found that LDs were directly sequestered by the *A. protothecoides* vacuole during the HA transition. This occurred via a microautophagy-resembling pathway that involved the protrusion of the vacuolar membrane rather than engulfment by double membrane structures such as autophagosomes. However, the vacuole was not large enough to entirely engulf the lipid bodies during early stages of HA transition. TEM images suggested that the cells might adopt two strategies to break down large LDs. The first step involves lipolysis, in which the large lipid bodies are disintegrated by LD-resident TAG lipases into many small-sized LDs. Alternatively, a portion of the large lipid bodies might protrude into the autophagic vacuole, be pinched off into the vacuolar lumen, and then be hydrolyzed by vacuolar lipase. The partial sequestration of organelles by the autophagic vacuole in yeast has been described previously for piecemeal microautophagy of the nucleus (PMN) (Roberts et al., 2003). In the second step, small LDs could be integrally incorporated into the central vacuole for autophagy degradation by inward invagination. This resembles lipid autophagy in yeast and is distinct from lipid metabolism in mammalian cells and plant seeds. The lipolysis and microautophagy pathways are therefore delicately orchestrated in *A. protothecoides* during the HA transition to satisfy the demands of lipid metabolism and energy supply.

### What induces autophagy during the HA transition?

Due to the inclusion of glucose, the heterotrophic cultivation medium used for *A. protothecoides*is relatively nutritious compared to the medium used for autotrophic growth. As a result, cells probably undergo carbon starvation upon transfer into glucose-free autotrophic media during the delay before a functioning chloroplast is established. Carbon starvation may serve as an inducer to trigger autophagy in a similar manner to the classic nitrogen starvation signal found in other organisms. Autophagy could be induced in *A. protothecoides* during the HA transition to allow cells to generate enough energy to propagate and to rebuild the photosynthetic machinery. Similar phenomena have been reported in other systems such as tobacco BY-2 cells and *Arabidopsis* suspension cells during sucrose starvation (Takatsuka et al., 2004; Rose et al., 2006). However, no evidence linking autophagy to lipid degradation was presented in those studies.

### Conflict of interest statement

The authors declare that the research was conducted in the absence of any commercial or financial relationships that could be construed as a potential conflict of interest.
