# Supplementary material for: Autophagy-like processes are involved in lipid droplet degradation in Auxenochlorella protothecoides during the heterotrophy-autotrophy transition
Source: Front Plant Sci. 2014 Aug 14;5:400. doi: 10.3389/fpls.2014.00400 (PMC4132264; doi:10.3389/fpls.2014.00400)
Supplement: Supplementary file 1 [file DataSheet1.DOCX]

***Supplementary Material***

**Autophagy-like processes are involved in lipid droplet degradation in *Auxenochlorella protothecoides* during the heterotrophy-autotrophy transition**

**Li Zhao, Junbiao Dai*, and Qingyu Wu***

MOE Key Laboratory of Bioinformatics, School of Life Sciences, Tsinghua University, Beijing, China

*** Correspondence:**

Junbiao Dai, MOE Key Laboratory of Bioinformatics and Center for Epigenetics and Chromatin,School of Life Sciences, Tsinghua University, Beijing,100084,China.

[jbdai@tsinghua.edu.cn](mailto:jbdai@tsinghua.edu.cn)

Qingyu Wu, MOE Key Laboratory of Bioinformatics, School of Life Sciences, Tsinghua University, Beijing, 100084, China.

[qingyu@mail.tsinghua.edu.cn](mailto:qingyu@mail.tsinghua.edu.cn)

Supplementary Table 1. Primers used to amplify *ApATG8* and *ApATG4* from an *A. protothecoides* cDNA library

| Primers | Sequences |
| --- | --- |
| ApATG8-F | ATGGTCCGCACCAAGACTTTC |
| ApATG8-R | CTAGCAGCCAAAGGTGCTCTC |
| ApATG4-F | ATGGGTGATCAGGATGCGCTGAGCAC |
| ApATG4-R | TCAGAGCATCTCCCAGCCAGAGCGAG |


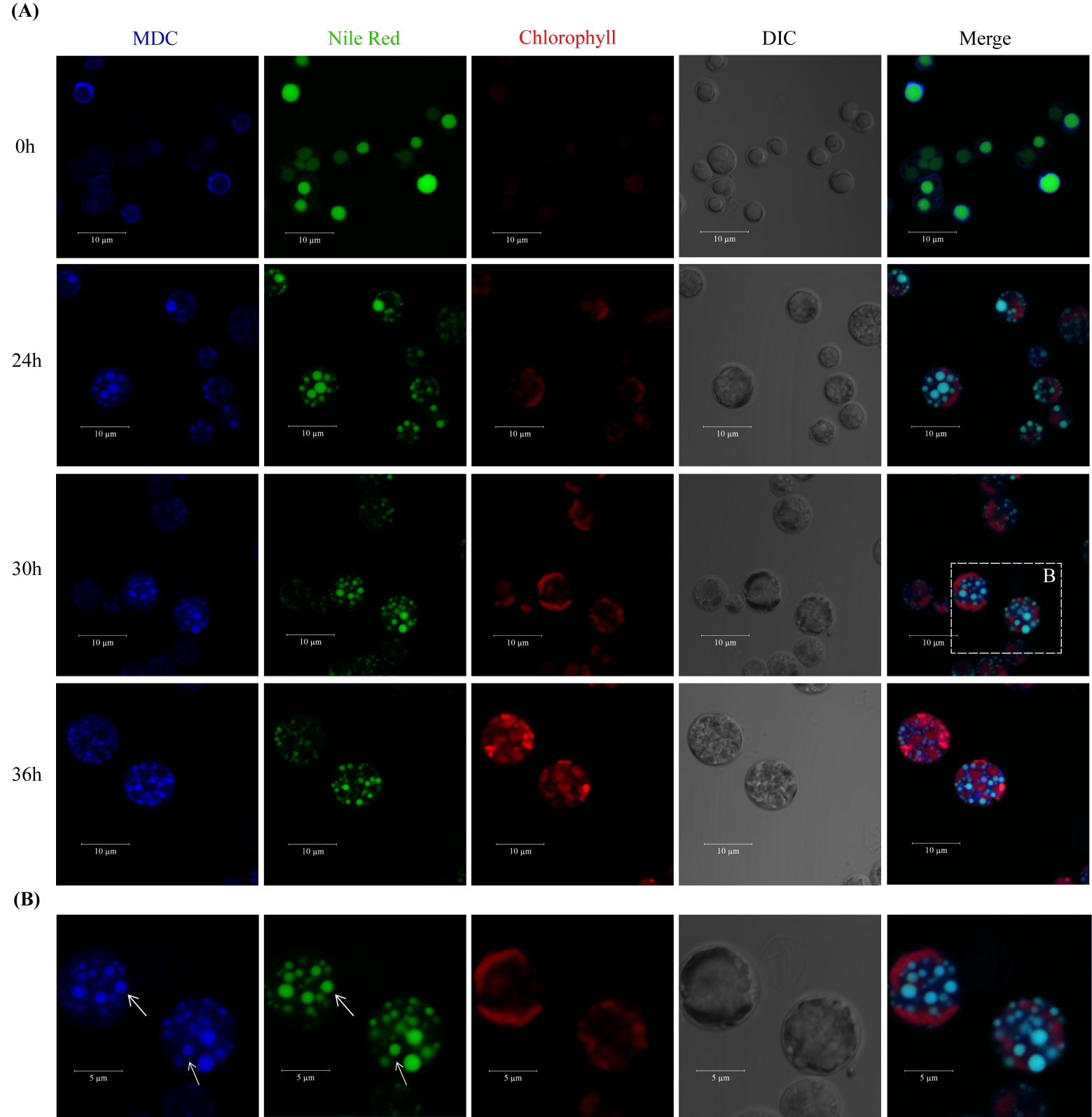


Supplementary Figure 1. Co-localization of MDC-labeled structures and lipid bodies in *A. protothecoides* during the HA transition. (A) Confocal microscope images of *A. protothecoides* cells stained with Nile red and MDC. Cells were harvested at the indicated time, stained with Nile red for 10min at 37°C, washed twice, and stained with MDC for 30min at 30°C. DIC, Differential interference contrast microscopy images. Scale bar, 10μm. (B) Enlargement of the 30h sample from (A) Arrows indicate co-localization of lipid bodies and MDC-labeled structures. Scale bar, 5 μm.
